# Radial Head Fractures in Adults: A Narrative Review

**DOI:** 10.7759/cureus.98559

**Published:** 2025-12-06

**Authors:** Ahmed Mohamed, Usman Fuad, Usama Farook, Alaa Elasad, Adham Elsayed, Ioannis P Pengas

**Affiliations:** 1 Trauma and Orthopaedics, Royal Cornwall Hospital, Truro, GBR; 2 General Practice, Zagazig University, Zagazig, EGY

**Keywords:** elbow fractures, elbow stability, mason classification, radial head fractures, radial head replacement

## Abstract

Radial head fractures are common injuries, particularly in adults, following falls onto an outstretched hand. These fractures are common in emergency departments and orthopedic clinics. The radial head is important for elbow stability, forearm rotation, and force transmission across the upper extremity. Understanding the anatomy, mechanisms of injury, classification systems, and treatment approaches is essential for healthcare providers managing these injuries. This narrative review synthesizes current evidence from peer-reviewed literature, clinical guidelines, and systematic reviews on radial head fractures, focusing on contemporary management strategies. We examine the epidemiology, clinical presentation, diagnostic evaluation, and management strategies for these injuries. We explore both conservative and surgical treatment options, discussing the indications for each approach and their associated outcomes. Additionally, this article addresses the complications that may arise following radial head fractures and provides insights into the rehabilitation protocols. The management of radial head fractures continues to evolve with advances in surgical techniques and implant technology, making it important for clinicians to remain informed about current best practices. This comprehensive review aims to provide a practical understanding of radial head fractures to assist healthcare providers in delivering optimal care.

## Introduction and background

Despite its small size, the radial head plays an important role in upper limb function. It contributes to elbow stability, allows for smooth rotation of the forearm, and helps transmit forces from the hand and wrist up through the elbow joint. When the radial head is fractured, these functions can be significantly compromised, leading to pain, restricted movement, and potential long-term disability if not properly managed [1].

Radial head fractures are common elbow fractures with an incidence of 2.5-2.8 per 10,000 person-years. They are the most common fractures around the elbow joint. The peak incidence occurs in adults aged 30-40 years. These injuries are uncommon in children. Women sustain radial head fractures approximately 1.3 times more frequently than men [2].

The management of radial head fractures has evolved considerably over the past few decades. Historically, prior to the 1980s, treatment approaches often involved simple immobilization for minimally displaced fractures or excision of the fractured radial head for comminuted injuries [3]. However, as our understanding of elbow biomechanics has improved, treatment strategies have become more sophisticated. Surgeons now recognize the importance of preserving the radial head whenever possible to maintain elbow stability and function. When preservation is not feasible, modern prosthetic replacements can restore anatomy and function more effectively than excision alone [4]. The evolution from radial head excision to contemporary arthroplasty techniques represents a paradigm shift in the management of complex radial head fractures, driven by biomechanical research and improved understanding of the radial head's role in elbow kinematics.

Understanding radial head fractures requires knowledge of several key aspects. First, the anatomy and biomechanics of the elbow joint provide a foundation for understanding how these injuries occur and why they affect function. Second, the various classification systems help guide treatment decisions by categorizing fractures based on their severity and associated injuries. Third, an accurate diagnosis through physical examination and imaging is essential for appropriate management. Finally, both conservative and surgical treatment options must be considered in the context of each patient's injury pattern, functional demands, and overall health status.

This narrative review aims to synthesize current evidence on the classification, diagnostic evaluation, and management strategies for radial head fractures, with particular emphasis on treatment decision-making, surgical techniques, and complication prevention. By providing a comprehensive overview of contemporary practice, this review seeks to guide clinicians in delivering evidence-based, patient-centered care for patients with radial head fracture.

## Review

Methods

This narrative review provides a comprehensive overview of the current knowledge and clinical practice regarding radial head fractures. A literature search was performed using PubMed, Embase, and Google Scholar databases. Search terms included combinations of "radial head fracture", "radial head arthroplasty", "Mason classification", "elbow trauma", "ORIF radial head", "radial head excision", "elbow instability", and "terrible triad injury". The search focused primarily on articles published between 2000 and 2025 to capture contemporary management strategies, though seminal historical papers were included to provide historical context. Articles were selected based on their relevance to clinical practice, including systematic reviews, meta-analyses, randomized controlled trials, prospective and retrospective cohort studies, technical reports, and expert consensus statements. Priority was given to Level I-III evidence, though case series and expert opinions were included when addressing uncommon complications or technical considerations. Only studies published in English were included. Studies on pediatric radial head fractures were excluded because this review focused specifically on adult injuries. The selected literature was synthesized to provide evidence-based recommendations for the diagnosis, treatment, and rehabilitation of radial head fractures in adults.

Anatomy and biomechanics

The radial head is a disk-shaped proximal end of the radius bone. It is located within the elbow joint and articulates with two important structures: the capitellum of the humerus and the radial notch of the ulna. The superior surface of the radial head is slightly concave and covered with articular cartilage, allowing it to glide smoothly against the capitellum during flexion and extension. The circumference of the radial head is also covered with cartilage, where it contacts the radial notch of the ulna during forearm rotation.

**Figure 1:**
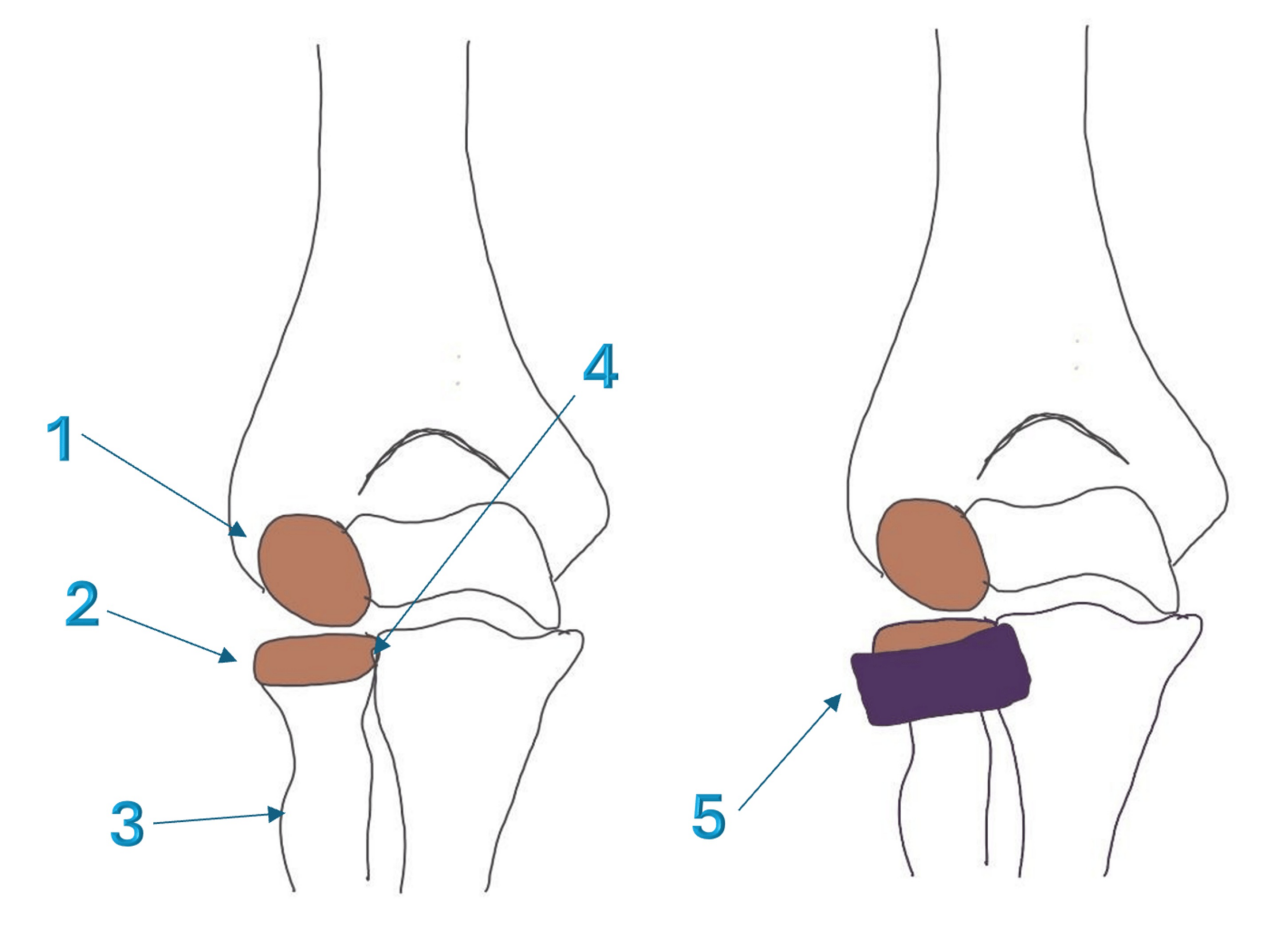
Anatomical relations of the radial head 1- Capitellum, 2- Radial head, 3- Radial tuberosity, 4- Proximal radio-ulnar joint, 5- Annular ligament Image credit:  Alaa Elasad (author); created using Infinite Painter application (https://www.infinitestudio.art/discover.php) and annotated using Microsoft Office 365 (Microsoft Corporation, Redmond, Washington, United States)

The radial head connects to the shaft of the radius through the radial neck, which is a narrower region just below the head. The radial tuberosity, where the biceps tendon inserts, is located just below the neck on the medial aspect of the radius. This anatomical relationship is important because injuries to the radial head can affect biceps function, and surgical approaches must avoid damaging the biceps insertion. Figure 1 explains all the important anatomical relations to the radial head.

Several ligaments provide stability to the elbow joint and work in conjunction with the radial head. The annular ligament wraps around the radial head and attaches to the ulna. It creates a ring that holds the radial head in place while allowing its rotation. The lateral collateral ligament complex provides important resistance to elbow instability. The medial collateral ligament on the opposite side also contributes to overall elbow stability. When this ligament is injured in combination with a radial head fracture, the elbow can become significantly unstable.

The radial head provides several biomechanical functions. It acts as a secondary stabilizer against valgus stress, particularly when the elbow is flexed. During daily activities, the radial head bears approximately 60% of the axial load of the elbow joint [5]. This load-sharing function protects the ulnohumeral joint from excessive stress. The radial head is essential for smooth forearm rotation. As the forearm pronates and supinates, the radial head rotates within the annular ligament, maintaining contact with the capitellum [6].

**Figure 2:**
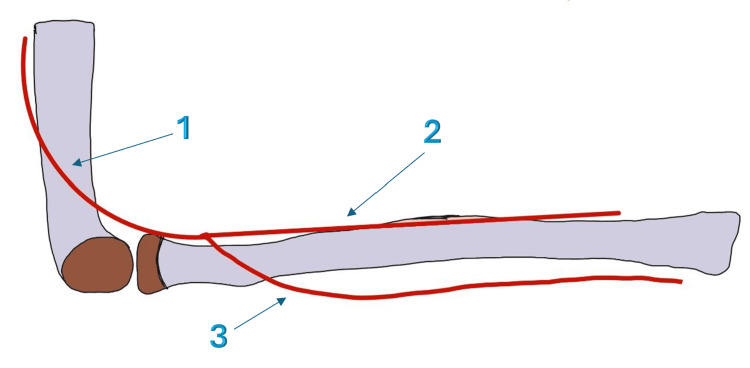
Anatomy of the posterior interosseous nerve 1- Radial nerve, 2- Superficial radial nerve, 3- Posterior interosseous nerve Image credit:  Alaa Elasad (author); created using Infinite Painter application (https://www.infinitestudio.art/discover.php) and annotated using Microsoft Office 365 (Microsoft Corporation, Redmond, Washington, United States)

Understanding the neurovascular structures around the radial head is critical for recognizing injury patterns and avoiding complications during surgical treatment. The posterior interosseous nerve (PIN) is the most important neural structure at risk during radial head fracture surgery (Figure 2). This nerve is a branch of the radial nerve that arises approximately at the level of the radiocapitellar joint. It wraps around the radial neck approximately 2-3 cm distal to the radial head. During surgical treatment, the nerve can be injured if the dissection extends too far distally. Injury results in finger and thumb extension weakness. The superficial branch of the radial nerve provides sensation to the dorsal hand and can be injured during lateral approaches, causing numbness [7].

The median and ulnar nerves are less commonly injured but should be assessed, particularly in radial head fractures associated with elbow dislocation. The radial recurrent artery supplies blood to the radial head and surrounding tissues. The blood supply to the radial head enters through small foramina in the radial neck. This can be disrupted by fractures or surgical dissection, increasing the risk of avascular necrosis [8].

Mechanism of injury and associated injuries

The most common mechanism of injury is falling onto an outstretched hand. When a person falls, they instinctively extend their arms and wrists to break the fall. The hand first contacts the ground, and the force of impact is transmitted through the wrist and forearm. As this force reaches the elbow, it creates an axial compression load on the radial head, which is pressed against the capitellum. If the force exceeds the strength of the bone, the radial head fractures. The specific fracture pattern depends on several factors, including the direction of the force, the position of the elbow at impact, and the quality of the bone. Less commonly, radial head fractures can result from direct trauma to the elbow, such as in motor vehicle accidents or industrial injuries. These high-energy mechanisms tend to produce more severe fractures and are more likely to be associated with other injuries [9].

Radial head fractures are the most common fractures around the elbow and are highly associated with other injuries. Studies showed that lateral collateral ligament injury and capitellar fractures are the most common associated injuries [1]. The combination of a radial head fracture, coronoid process fracture, and posterior elbow dislocation is known as the terrible triad injury due to its association with poor outcomes if not properly treated.

The Essex-Lopresti injury involves a radial head fracture combined with disruption of the interosseous membrane between the radius and ulna and injury to the distal radioulnar joint (DRUJ). This injury pattern is frequently missed in the acute setting because wrist symptoms may be subtle or overshadowed by the more obvious elbow pain and swelling. Failure to recognize an Essex-Lopresti injury can lead to devastating long-term consequences, including proximal migration of the radius, chronic wrist pain from ulnocarpal impaction, loss of grip strength, and severe functional disability. Clinicians must maintain a high index of suspicion and carefully examine the entire forearm and wrist in patients with radial head fractures, particularly those resulting from high-energy mechanisms. Early recognition and appropriate treatment are critical for preventing chronic disability [10].

Classification systems

Several classification systems have been developed to categorize these fractures. The Mason classification, originally described in 1954, is the most widely used classification system for radial head fractures [3]. This system has been modified over time to improve its clinical applicability and address limitations of the original scheme. The most commonly used modification was proposed by Hotchkiss in 1997 [11] and further refined by van Riet and Morrey [12], who added Type IV fractures to account for radial head fractures associated with elbow dislocation (Table 1).

**Table 1:** Mason classification for radial head fractures

Type	Description	Characteristics	Treatment Implications
Type I	Non-displaced or minimally displaced	No mechanical block to forearm rotation	conservative management
Type II	Displaced fracture	Greater than 2mm displacement, marginal fragment involvement, possible mechanical block	Amenable to surgical fixation if there is mechanical block
Type III	Comminuted fracture	Severe comminution involving entire radial head	Not reconstructable, requires excision or replacement
Type IV	Associated dislocation	Any radial head fracture with elbow dislocation	Indicates significant soft tissue injury, requires comprehensive assessment

Clinical presentation and physical examination

**Figure 3:**
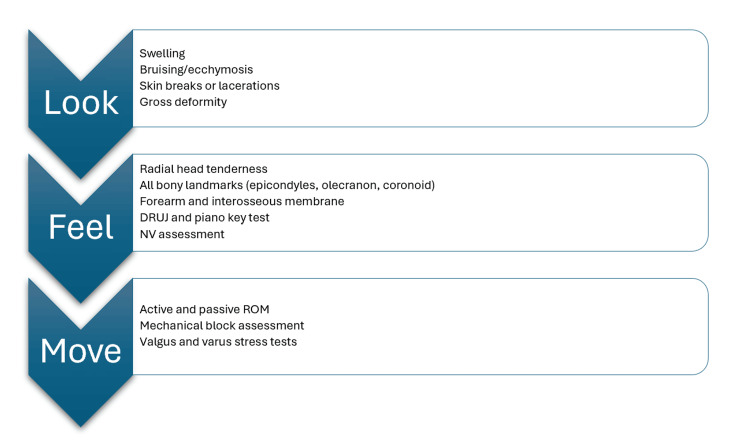
Examination findings in radial head fractures Image credit:  Alaa Elasad (author); created using Microsoft Office 365 (Microsoft Corporation, Redmond, Washington, United States) DRUJ: distal radio-ulanr joint; ROM: range of motion; NV: neurovascular

Clinical presentation findings can be divided into three main categories: (i) look, (ii) feel, and (iii) move, for completeness (Figure 3).

Look

Physical examination begins with a careful inspection of the affected upper limb. The examiner should look for swelling or bruising around the elbow joint. The skin should be examined carefully for any breaks, lacerations, or abrasions, as these may indicate an open fracture requiring urgent intervention. Any gross deformity should raise suspicion for associated injuries, such as elbow dislocation or more complex fracture patterns.

Feel

Palpation is a critical component of the examination and should be performed systematically by palpating all landmarks around the elbow. Direct tenderness of the radial head is a sensitive finding in radial head fractures. Care should be taken not to cause too much pain to the patient, especially with acute fractures. Palpating the medial and lateral epicondyles, olecranon, and coronoid process is also important, as it may indicate associated fractures or ligamentous injuries.

The entire forearm should be palpated from the elbow to the wrist. Tenderness along the interosseous membrane or at the DRUJ may indicate an Essex-Lopresti injury. A positive piano key test at the wrist, where the ulnar head can be depressed like a piano key, is another symptom of DRUJ instability. Essex-Lopresti injury results in longitudinal forearm instability, where the radius migrates proximally, leading to chronic wrist pain (as the ulna will be impacted against the carpal bones), grip weakness, and restricted forearm rotation if unrecognized. Early recognition of an Essex-Lopresti lesion is crucial, as a missed diagnosis can lead to painful chronic instability and loss of forearm function.

A thorough neurovascular examination is essential. The radial nerve (and its posterior interosseous component) should be carefully assessed by checking for wrist, thumb, and finger extension, as weakness in these movements suggests radial nerve injury. Sensation should be tested in the distribution of the radial nerve, including the first dorsal web space. The ulnar and median nerves should also be examined, and both motor and sensory functions should be tested. Vascular assessment included checking the radial and ulnar pulses, capillary refill in the fingertips, and comparing the temperature and color of the injured extremity with the uninjured side.

Move

The range of motion (ROM) assessment of the forearm includes four movements. Physicians should assess flexion, extension, pronation, and supination. Flexion and extension should be first performed by asking the patient to flex and extend the elbow as much as they can tolerate. Then, the examiner should assess pronation and supination with the elbow held at 90 degrees of flexion. This position isolates forearm rotation from the shoulder motion. The ROM should be assessed actively and passively to avoid excessive pain. Examiners should look at the extent of the ROM as well as any mechanical block. Patients with displaced fractures often have a mechanical block to rotation, where they can move through part of the arc but then experience a hard stop. This mechanical block indicates that a fracture fragment impinges on adjacent structures, requiring surgical intervention. In some cases, especially with acute fractures, the elbow joint is significantly swollen. Elbow swelling can create a pseudo-mechanical block. Therefore, a reliable movement examination of the elbow should not be performed acutely, and patients should be reviewed in the fracture clinic after approximately a week to allow the swelling to subside and to detect if there is a true mechanical block. Moreover, testing for ligamentous instability by performing varus and valgus stress tests is important.

Diagnostic imaging

Plain radiographs are the initial imaging study for suspected radial head fractures. Standard elbow radiographs include anteroposterior, lateral, and oblique views. The radiocapitellar view is a specialized oblique view that profiles the radial head without coronoid overlap. This view helps in detecting very small fractures. The presence of a posterior fat pad sign or elevation of the anterior fat pad indicates elbow joint effusion, which is common in intra-articular fractures [13].

Computed tomography (CT) is increasingly important for evaluating radial head fractures. CT scans provide detailed information about fracture patterns, fragment number, size, displacement, articular surface involvement, and associated fractures. CT is helpful for complex fractures where surgical fixation is being considered. The decision to obtain a CT scan should be based on radiographic findings and the anticipated treatment [14].

Magnetic resonance imaging (MRI) is not routinely used for radial head fractures, but it can be valuable in identifying associated soft tissue injuries, including ligamentous tears and cartilage injuries

Treatment options

Conservative Management

Non-displaced or minimally displaced radial head fractures are often treated conservatively. The goal of conservative treatment is to control pain and swelling. Immobilization in a sling should be for a few days till pain subsides to avoid elbow stiffness. The next stage should be gentle ROM exercises until physicians confirm clinical and radiological healing. Most non-displaced fractures heal within six to eight weeks. Then, weight-bearing exercises can begin to regain the lost strength. During this treatment journey, follow-up radiographs are obtained to ensure that the fracture is not displaced [15].

Surgical Management

Surgical intervention is indicated for displaced fractures, comminuted fractures, fractures with mechanical block to motion, and fractures associated with elbow instability. The specific surgical approach depends on the fracture pattern and associated injuries. Treatment options include open reduction and internal fixation (ORIF), radial head excision, and radial head replacement.

ORIF is appropriate for fractures involving a substantial portion of the articular surface that are reconstructable. Approaches to the radial head depend on the fracture pattern, displacement, and associated injuries. Two approaches are commonly used to access radial head fractures. The lateral (Kocher) approach, between the anconeus and extensor carpi ulnaris, is the most common and offers excellent access to the radial head and neck while minimizing the risk to the posterior interosseous nerve (PIN) if dissection is kept posterior and the forearm is pronated. The Kaplan approach between the extensor digitorum communis and extensor carpi radialis brevis provides more anterior exposure for anterior fractures but carries a slightly higher risk of PIN injury. Fixation options vary with fracture type. Small, simple fractures can be stabilized with headless screws, whereas comminuted patterns may require low-profile radial head plates contoured to the lateral surface of the proximal radius [16-18]. Implants should be placed within the safe zone (a 90°-110° non-articulating arc on the lateral and slightly posterior aspect of the radial head) to prevent impingement on the proximal radioulnar joint during rotation [19]. A careful approach to selection, implant positioning, and protection of neurovascular structures is essential for optimal outcomes.

Radial head excision involves removing the fractured radial head entirely. It is generally reserved for severely comminuted fractures in elderly, low-demand patients without associated instability or ligamentous injury. It is contraindicated in the presence of elbow or forearm instability, such as disruption of the interosseous membrane, medial collateral ligament, or coronoid process fractures, as excision in these cases can lead to proximal radial migration and ulnocarpal impaction. The excision should be performed just distal to the articular margin of the head, preserving the radial neck and annular ligament to maintain elbow stability. Overresection must be avoided to prevent valgus instability and radiocapitellar incongruity. Although radial head excision can relieve pain and restore motion in low-demand patients, it is rarely used in modern practice because radial head replacement offers better stability and functional outcomes in most unstable or complex fracture scenarios [20-22].

Radial head replacement (arthroplasty) is indicated for severely comminuted or non-reconstructable fractures (Mason type III or IV) in active patients, particularly when associated with elbow instability or ligamentous injury. The goal is to restore radiocapitellar contact, maintain elbow stability, and prevent the proximal migration of the radial head. Proper implant sizing and positioning are critical for successful outcomes. Overlengthening can lead to limited motion, pain, and capitellar erosion, whereas undersizing may cause instability and proximal migration. Modern prostheses are modular metallic implants that allow the adjustment of head height and diameter to match the patient’s anatomy. The stem may be press-fit or cemented, depending on the bone quality and implant design. When correctly implanted, radial head replacement provides excellent pain relief and stability, facilitating early mobilization and improved functional outcomes compared with excision in unstable or complex fractures [17,21,23,24].

Complications

Several complications may occur after radial head fractures. Elbow stiffness is the most common complication, occurring in approximately 20-40% of patients following radial head fractures, with higher rates observed after surgical treatment and complex injury patterns. The elbow joint is particularly prone to stiffness after injury, especially if it is immobilized for prolonged periods. Loss of extension is often more problematic than loss of flexion. Prevention through early mobilization is the best strategy, but once stiffness develops, treatment can be challenging [25].

Post-traumatic osteoarthritis can develop in the elbow joint after radial head fractures, particularly if the articular surface is not restored anatomically or in the case of comminuted fractures. The reported incidence ranges from 15% to 30% of patients. Patients may experience chronic pain, swelling, and limited motion. Mild arthritis can often be managed with activity modification, anti-inflammatory medications, and steroid injections; however, severe arthritis may eventually require elbow joint replacement [26].

Nerve injuries associated with radial head fractures may occur at the time of injury or as a complication of surgical management. The PIN is at the highest risk of injury. PIN injury occurs in approximately 2-10% of radial head fractures, with higher rates (up to 15-20%) following surgical intervention. Traction, compression from hematoma or swelling, and direct surgical manipulation during exposure can all lead to temporary neuropraxia. Clinically, this presents as weakness or loss of active extension of the fingers and thumb, while wrist extension is preserved due to the intact extensor carpi radialis longus, which is supplied by the main radial nerve before it splits into the superficial radial nerve and PIN. Neuropraxia requires up to 12 weeks for full recovery. If nerve injury is detected after surgery, nerve conduction studies (NCS) and electromyography (EMG) can help differentiate between a simple traction injury and a complete injury of the nerve, providing prognostic information and allowing early referral to the nerve injury units [27].

Heterotopic ossification (HO) refers to the abnormal formation of mature lamellar bone within soft tissues. The incidence of HO after radial head fractures ranges from 5% to 15%, with significantly higher rates (20-30%) following high-energy trauma, fracture-dislocations, delayed surgical intervention, or prolonged surgery. Around the elbow, it most commonly develops in the anterior or posterior capsule or within the brachialis or triceps muscles, leading to pain, swelling, and progressive loss of motion. HO results from inappropriate differentiation of mesenchymal stem cells into osteogenic cells triggered by local inflammation and tissue hypoxia. Prevention focuses on minimizing local inflammation and promoting early gentle mobilization after surgery or injury. Pharmacological prophylaxis should be considered in high-risk patients. This commonly includes non-steroidal anti-inflammatory drugs (NSAIDs). Once HO matures and causes clinically significant stiffness or functional limitations, surgical excision may be indicated. Excision should be delayed until radiographic and clinical evidence of maturity (usually 6-12 months post injury) to minimize recurrence. Postoperative prophylaxis with NSAIDs or radiotherapy is often employed to reduce the risk of recurrence after excision [28-30].

Instability of the elbow can occur if associated ligamentous injuries are not recognized and treated, or if the radial head is excised in the setting of ligament injury. The incidence of chronic elbow instability following radial head fractures is approximately 5-10%, predominantly in cases where associated injuries (medial collateral ligament, coronoid fractures) are missed or inadequately treated. Patients with instability may experience pain, weakness, and a sensation of the elbow giving way during activities. Treatment may require ligament reconstruction and, in some cases, radial head replacement if the radial head has been previously excised [31].

Hardware-related complications can occur after surgical fixation because the radial head is very close to the skin. Hardware complications occur in approximately 10-20% of cases treated with ORIF. Symptoms include symptomatic prominent hardware, mechanical impingement, and loosening. Screws or plates may become prominent and cause irritation or limit motion. Hardware removal may be necessary in some cases, including skin-threatening cases, due to prominent metalwork, intra-articular metalwork, loosening, or infection. Infection is always a risk with any surgery, although it is uncommon with elbow procedures, occurring in approximately 1-3% of cases [32,33].

Nonunion, where the fracture fails to heal, is uncommon with radial head fractures, occurring in approximately 2-5% of cases. Risk factors include poor blood supply, inadequate fixation, and smoking. Treatment requires revision surgery with bone grafting and stable fixation [34,35].

Rehabilitation

Rehabilitation after radial head fracture is essential for optimal outcomes. The goals of rehabilitation are to restore the ROM, strength, and function while minimizing pain and preventing complications. The specific rehabilitation protocol depends on the fracture severity and treatment method used.

For conservatively treated fractures, rehabilitation begins with gentle ROM exercises once the acute pain subsides. Patients are encouraged to perform elbow flexion and extension exercises as well as forearm rotation exercises several times daily. The exercises should be performed within a comfortable range and gradually progressed as tolerated. Ice can be applied after exercise to control swelling. As motion improves, strengthening exercises are introduced using light weights and elastic bands. Strengthening should focus on all muscle groups around the elbow, including the biceps, triceps, and forearm muscles [4].

For surgically treated fractures, the rehabilitation protocol depends on the stability of fixation. When fractures are well fixed, early motion can begin within a few days of surgery. Surgeons should provide specific guidelines regarding motion restrictions and weight-bearing limitations. Continuous passive motion machines are sometimes used in the early postoperative period to maintain motion while minimizing active muscle forces across the fracture. This should be followed by manual therapy and exercises to improve motion [36].

Attention should be paid to returning to functional activities and work. This includes activities that simulate daily tasks and work-related or sports-specific activities. Contact sports and activities that place high demands on the elbow should be avoided until adequate healing occurs and the patient regains full motion and strength. The timeline varies but ranges from three to six months for most patients [37].

## Conclusions

Radial head fractures are common injuries that significantly affect elbow function. Successful management requires accurate diagnosis and recognition of associated injuries. Radial head preservation through fixation should be the primary goal. When preservation is not feasible, arthroplasty is preferred over excision in active patients. Early mobilization is critical to prevent stiffness. CT imaging helps guide surgical planning for complex fractures. Treatment decisions must be individualized based on fracture pattern, patient activity level, and associated injuries. Tailored rehabilitation protocols are essential for optimal recovery.

Important knowledge gaps remain in the literature. High-quality studies comparing fixation versus arthroplasty for Mason Type III fractures are lacking. Long-term outcomes of different prosthetic designs remain unclear. Evidence-based prophylaxis strategies for heterotopic ossification are not established. Standardized rehabilitation protocols are absent. Future research should address these gaps through prospective trials. Patient-reported outcome measures should be incorporated. As surgical techniques continue to advance, clinicians must stay informed about evolving evidence. Focus should remain on accurate diagnosis, anatomic restoration, early mobilization, and recognition of associated injuries.
